# Direct, indirect and total effectiveness of bivalent HPV vaccine in women in Galicia, Spain

**DOI:** 10.1371/journal.pone.0201653

**Published:** 2018-08-03

**Authors:** M. Jesus Purriños-Hermida, María Isolina Santiago-Pérez, Mercedes Treviño, Rafaela Dopazo, Angelina Cañizares, Isolina Bonacho, Matilde Trigo, M. Eva Fernández, Ana Cid, David Gómez, Patricia Ordóñez, Amparo Coira, M. J. Armada, Magdalena Porto, Sonia Perez, Alberto Malvar-Pintos, on behalf of the HPV Vaccine Impact Surveillance Working Group of Galicia

**Affiliations:** 1 Servizo de Epidemioloxía, Dirección Xeral de Saúde Pública, Consellería de Sanidade, Xunta de Galicia, Santiago de Compostela, Spain; 2 Microbiology Department, University Hospital of Santiago, Santiago de Compostela, Spain; 3 Gynecology Department, University Hospital of Santiago, Santiago de Compostela, Spain; 4 Microbiology Department, University Hospital of A Coruña, A Coruña, Spain; 5 Gynecology Department, University Hospital of A Coruña, A Coruña, Spain; 6 Microbiology Department, University Hospital of Pontevedra, Pontevedra, Spain; 7 Gynecology Department, University Hospital of Pontevedra, Pontevedra, Spain; 8 Microbiology Department, University Hospital of Ourense, Ourense, Spain; 9 Gynecology Department, University Hospital of Ourense, Ourense, Spain; 10 Microbiology Department, University Hospital of Ferrol, Ferrol, Spain; 11 Microbiology Department, University Hospital of Lugo, Lugo, Spain; 12 Fingoy Primary Care Department, Sanitary Area of Lugo, Lugo, Spain; 13 Gynecology Department, University Hospital of Vigo, Vigo, Spain; 14 Microbiology Department, University Hospital of Vigo, Vigo, Spain; 15 Galicia Sur Health Research Institute, Vigo, Vigo, Spain; Rudjer Boskovic Institute, CROATIA

## Abstract

Bivalent human papillomavirus (HPV) vaccine was incorporated into the childhood vaccination calendar in Galicia, Spain in 2008. The objectives of this study were to estimate direct, indirect and total effectiveness of HPV vaccine and to identify sexual habits changes in the post-vaccination period in Galicia, Spain.Endocervical scrapings of 745 women attending 7 Health Areas of the Galician Public Health Service were collected in the post-vaccination period, from 2014–2017. Two groups were studied: women born between 1989 and 1993 (n = 397) and women born in 1994 or later (n = 348). Twelve high-risk human papillomavirus (HR-HPV) genotypes were detected by Cobas® 4800 HPV test (Roche Diagnostics, Mannheim, Germany). The Linear Array® HPV Genotyping Test (Roche Diagnostics) was used for HR-HPV genotype detection other than HPV 16/18. Information about sexual habits was collected by a self-filled questionnaire. Post-vaccination data were compared to previously published pre-vaccination data obtained between 2008 and 2010 in Galicia from women of the same age (18–26 years old, n = 523). The Stata 14.2 software was employed for statistical analyses.Data from 392 unvaccinated and 353 vaccinated women were compared. For unvaccinated and vaccinated women, HPV 16/18 prevalence was 9.2% and 0.8%, respectively, and HPV 31/33/45 prevalence was 8.4% and 1.1%, respectively. Direct, indirect and total effectiveness of the HPV vaccine were (%, 95% CI): 94 (72−99), 30 (-11−56) and 95 (79−99), respectively, for HPV 16/18 and 83 (46−94), -10 (-88−33) and 84 (54−94), respectively, for HPV 31/33/45. The number of women with first intercourse before 17 years old and 3 or more sexual partners along life was higher in the post-vaccination period (*p* < 0.05). A positive impact of bivalent HPV vaccine was observed, both on direct and cross protection. Sexual habits could have changed in the post-vaccination period.

## Introduction

Persistent infection of high-risk human papilloma virus (HR-HPV) is thought to be responsible for about 100% of cervical cancer cases [1]; this cancer ranks second among malign neoplasia in women worldwide [2] and causes, according to WHO estimates, 274,000 deaths per year [1]. In addition, HR-HPV are included among the etiological agents of 78% of vagina cancer and most of their precursor lesions in young women [3], 31% of oropharyngeal cancer, 15% of vulva cancer, 4.3% of oral cavity cancer and 4.6% of laryngeal cancer [4].

To reduce the incidence of these pathologies, mainly of cervical cancer, several vaccines against HPV have been developed. HPV 16 and 18 are responsible for approximately 70% of world cervical cancer [1,5] and HPV 6 and 11 genotypes are responsible for 90% of genital warts. This is the reason why the first commercial vaccines were developed against these genotypes: a bivalent vaccine against HPV 16 and 18 (with some cross-protection against HPV 31, 33 and 45) and a quadrivalent vaccine against genotypes HPV 6, 11, 16 and 18. Subsequently, in 2014, the FDA approved a new vaccine against 9 genotypes (HPV 6, 11, 16, 18, 31, 33, 45, 52 and 58), which is licensed in Europe and now commercially available in Spain. Bivalent and quadrivalent vaccines are very effective and safe vaccines [6–9].

In Galicia, the bivalent vaccine was introduced in the official immunization schedule for girls at the end of 2008. Three doses (0–1–6 months) were administered to girls born after the 1st of January of 1994 when they were 14 years old. In March 2014, there was a change to the two-dose schedule (0–6 months) authorized by the Spanish Agency of Medicines and Medical Devices. In January of 2016, the vaccination of 12 years old girls was introduced.

In Galicia, the pre-vaccination prevalence of HR-HPV genotypes was estimated to be around 10% in female general population, 19% in women 16–19 years old and 23% in women 20–24 years old. The most frequent genotype was HPV 16, with a prevalence of 3.5% [10].

In different countries, the introduction of HPV vaccination programs produced a decrease in the prevalence of HPV genotypes targeted by the vaccine (HPV 16 and 18) [11,12]. This achievement is considered an intermediate step to reduce the incidence of HPV related cancer. The effect of vaccination programs depends on vaccine use recommendations, implementation strategies (with or without catch up) and the achieved coverage.

Therefore, in order to evaluate the vaccine effectiveness and its impact on our population, it is essential to compare pre and post vaccine HPV prevalence. As HPV transmission route is sexual, this comparison should take into account any change in women's sexual behavior.

The objective of this paper was to estimate direct, indirect and total effectiveness of bivalent HPV vaccine and to identify changes in sexual habits after the introduction of the vaccination program in Galicia, Spain.

## Materials and methods

### Study population

In the post-vaccination period, the study population comprised women of 18–26 years old, born in 1989 or later, living in Galicia (Spain), attending a primary care center, gynecology department or family counseling center belonging to the Galician Public Health Service from 2014 to 2017. The exclusion criteria considered were: no previous intercourse with penetration, current consultation for a sexually transmitted infection (including HPV related lesion follow-up), pregnancy, giving birth in the previous 40 days, hysterectomy, psychiatric disorders or inability to fill in a self-filled questionnaire.

Twenty-three collaborators working throughout the seven Health Areas of Galicia (Spain) were selected by convenience criteria in order to cover the entire territory. Women were offered to participate following an established system based on appointment schedule and year of birth. Two cohorts were established according to the HPV vaccine recommendation stated by 2008 official immunization schedule in Galicia, Spain: women born between 1989 and 1993 (older cohort) and women born in 1994 or later (younger cohort). Collaborators were asked to include the same number of women from both groups (S1 Appendix). This patient recruitment continues today in an ongoing study (2014–2019).

During this post-vaccination period, HPV detection cervical scrapings were collected in ThinPrep PreservCyt Solution (Hologic, Marlborough, MA, USA) in 6 Health Areas and in BD SurePath™ Collection Vial (Becton Dickinson, New Jersey, USA) in one Health Area. Twelve HR-HPV genotypes were detected by Cobas® 4800 HPV test (Roche Diagnostics, Mannheim, Germany): HPV 16, 18, 31, 33, 35, 39, 45, 51, 52, 56, 58, 59, 66 and 68. The Linear Array® HPV Genotyping Test (Roche Diagnostics, Mannheim, Germany), which identifies eighteen HR-HPV (16, 18, 26, 31, 33, 35, 39, 45, 51, 52, 53, 56, 58, 59, 66, 68, 73 and 82), was used in case of HR-HPV genotype detection other than HPV 16/18.

On the other hand, in the pre-vaccination period (2008–2010), the study population comprised women of 18–26 years old, living in Galicia (Spain) and attending the Galician Public Health Service at Pontevedra, which is one of the seven Health Areas of Galicia (Spain). The exclusion criteria were the same as in the post-vaccination period. The HR-HPV prevalence observed in these patients were previously published [10]. In this period, the HPV detection was done using ThinPrep PreservCyt Solution (Hologic), Cobas® Amplicor HPV test (Roche Diagnostics), which detects eleven HR-HPV genotypes (HPV 16, 18, 31, 33, 35, 39, 45, 51, 52, 56, 58, 59 and 68). The Linear Array® HPV Genotyping Test (Roche Diagnostics) was utilized in case of a positive result.

In both periods, sexual habits information was collected by a self-filled written questionnaire (S2 and S3 Appendixes).

### Vaccination status evaluation

The vaccination status was self-reported and also extracted from the electronic clinical history. Women who received at least two HPV vaccine doses were considered as vaccinated. Women who did not received any dose of HPV vaccine were considered unvaccinated. Women who received a single dose of vaccine were also excluded from the analysis. All women from the pre-vaccination period were unvaccinated. For data comparison between vaccinated and unvaccinated women, the vaccination status was considered as stated in the electronic clinical history. When this information was not registered, the vaccination status was considered as stated in the questionnaire. When no information was available, the women was considered as unvaccinated.

### HPV genotyping

Detection of HPV 16 and/or 18 was assigned to the HPV 16/18 group. Detection of at least one of the three following genotypes HPV 31, 33, 45 was assigned to the HPV 31/33/45 group. Detection of any HR-HPV genotype different than HPV 16/18/31/33/45 was assigned to the other HR-HPV group. Detection of genotypes pertaining to more than one HPV group in the same sample was considered as a positive result in the corresponding HPV groups.

### Statistical analysis

The prevalence and the 95% confidence interval (95% CI) were calculated for each HPV genotype group. In case of detection of any HR-HPV, other than HPV 16/18, that could not be analyzed by the Linear Array test, the assignment to the HPV 31/33/45 group or to the other HR-HPV group was performed using the system of chained equations (multiple imputation, MICE) by logistic regression [13].

For each HPV genotype group, vaccine effectiveness was estimated as 1 –prevalence ratio (1 –PR). PR was calculated using a Poisson regression model considering the HPV status (positive or negative) as the dependent variable and the groups to be compared as independent variable: (1) vaccinated *vs*. unvaccinated women from post-vaccination period (direct effectiveness), (2) unvaccinated women from post-vaccination period *vs*. women from pre-vaccination period (indirect effectiveness), (3) vaccinated women from post-vaccination period *vs*. women from pre-vaccination period (total effectiveness), (4) women from post-vaccination period *vs*. women from pre-vaccination (overall effectiveness). The model was estimated unadjusted and adjusting by age group (18–20, 21–23, 24–26), by first intercourse > 16 years old, 3 or more partners along life, and 2 or more partners in the last year. In the post-vaccination period, a comparison was also performed by inclusion year: first biennium (2014–15) and second biennium (2016–17). The variance was estimated using the Huber and White sandwich estimator.

Epidemiological data were compared by vaccination status and time period. The qualitative variables were compared using Fisher's exact test. The concordance between two methods of vaccination status estimation was measured by Cohen's kappa coefficient.

To assess the statistical significance, a level of significance of 5% was considered (p < 0.05). The Stata 14.2 software was used for statistical analysis.

### Ethics statement

This study received approval from the Ethics Committee of Clinical Investigation of Galicia (Santiago de Compostela, Spain). All study participants were informed about the purpose of the survey and were asked to sign a consent form before taking part in the study. Confidentiality was ensured during data collection and subsequent publication of the results.

## Results

### Epidemiological characteristics of the population included in post-vaccination period

A total of 745 women (average age 21.7 years ± 2.2 SD) from all Health Care Areas of Galicia were included in the post-vaccination period: 447 (60%) in the first biennium (2014–2015) and 298 (40%) in the second biennium (2016–2017). The older cohort (53.3%) was born between 1989 and 1993. The younger cohort (46.7%) was born in 1994 or later. Eighty two percent was born in Galicia. Fifty seven percent had their first sexual relationship before age 17 and 71% declared to have had a sexual partner in the last year. The average age at the first intercourse was 16.4 years ± 1.7 SD. The vaccination coverage information was available in the electronic clinical history in 480 cases. It was only available in questionnaires in 209 cases and it was not available in 56 cases. The global vaccination coverage was 47% (10% with two doses and 90% with three doses). The vaccination coverage was 43% and 53% in the first and second biennium, respectively. A 96% concordance (Kappa 0.90) was observed between women knowledge about their vaccination status and the information registered in the clinical history.

Table 1 shows demographic characteristics and sexual habits of unvaccinated and vaccinated women, belonging to the post-vaccination period. The average age was 22.9 years for unvaccinated women *vs*. 20.4 for vaccinated women. The number of partners along life was 2 or more for 84.5% of unvaccinated women *vs*. 5 or less in 83.3% of vaccinated women (*p* = 0.022). The proportion of women having their first sexual relationship before age 17 was 54% in unvaccinated women *vs*. 61% in vaccinated women (*p* = 0.022).

**Table 1:** Post-vaccination period (2014–17): Characteristics of unvaccinated and vaccinated women.

	Unvaccinated		Vaccinated		*p* value
	n	%	n	%	
**Age**	392		353		
** 18–20 years old**	40	10.2	198	56.1	*< 0.001
** 21–23 years old**	187	47.7	128	36.3	
** 24–26 years old**	165	42.1	27	7.7	
** Average age: Years (SD)**		22.9 (1.9)		20.4 (1.9)	
**Birthplace**	386		352		
** Galicia**	301	78.0	301	85.5	*0.024
** Rest of Spain**	22	5.7	16	4.6	
** Other countries**	63	16.3	35	9.9	
**Arrival at Galicia**	58		35		
** After first intercourse**	25	43.1	1	2.9	*< 0.001
** Before first intercourse**	33	56.9	34	97.1	
**Age at first intercourse**	387		351		
** ≤ 16 years old**	209	54.0	215	61.3	0.053
** > 16 years old**	178	46.0	136	38.8	
** Average age: Years (SD)**		16.5 (1.9)		16.2 (1.6)	
**Number of partners along life**	384		352		
** 1**	60	15.6	83	23.6	*0.022
** 2**	84	21.9	84	23.9	
** 3–5**	158	41.2	126	35.8	
** > 5**	82	21.4	59	16.8	
**Number of partners in the last year**	375		340		
** None**	11	2.9	15	4.4	0.638
** 1**	269	71.7	236	69.4	
** 2**	53	14.1	54	15.9	
** ≥ 3**	42	11.2	35	10.3	

SD: Standard deviation.

* *p* < 0.05, statistically significant

### Epidemiological comparison with pre-vaccination period

Table 2 shows demographic characteristics and sexual habits of the studied population in the post-vaccination period in comparison with a population of similar age range studied in the pre-vaccination period. The proportion of women having 3 or more partners along life was 47.2% in pre-vaccination period *vs*. 57.8% of women studied in the post-vaccination period (*p* < 0.001). The proportion of women having their first sexual relationship before age 17 was 36.9% in the pre-vaccination period *vs*. 57.5% in the post-vaccination women (p < 0.001).

**Table 2:** Characteristics of women in the pre-vaccination (2008–10) and post-vaccination period (2014–17).

	Pre-vaccination period		Post-vaccination period		*p* value
	n	%	n	%	
**Age**	523		745		
** 18–20 years old**	188	36.0	238	32.0	*< 0.001
** 21–23 years old**	161	30.8	315	42.3	
** 24–26 years old**	174	33.3	192	25.8	
** Average age: Years (SD)**		21.9 (2.6)		21.7 (2.2)	
**Birthplace**	522		738		
** Galicia**	455	87.2	602	81.6	*0.028
** Rest of Spain**	19	3.6	38	5.2	
** Other countries**	48	9.2	98	13.3	
**Arrival at Galicia**	46		93		
** After first intercourse**	23	50.0	26	28.0	*0.014
** Before first intercourse**	23	50.0	67	72.0	
**Age at first intercourse**	523		738		
** ≤ 16 years old**	193	36.9	424	57.5	*< 0.001
** > 16 years old**	330	63.1	314	42.6	
** Average age: Years (SD)**		17.2 (2.0)		16.4 (1.7)	
**Number of partners along life**	487		736		
** 1**	147	30.2	143	19.4	*< 0.001
** 2**	110	22.6	168	22.8	
** 3–5**	157	32.2	284	38.6	
** > 5**	73	15.0	141	19.2	
**Number of partners in the last year**	487		715		
** None**	20	4.1	26	3.6	0.227
** 1**	364	74.7	505	70.6	
** 2**	66	13.6	107	15.0	
** ≥ 3**	37	7.6	77	10.8	

SD: Standard deviation.

* *p* < 0.05, statistically significant.

### Prevalence of HPV genotypes

**Fig 1:**
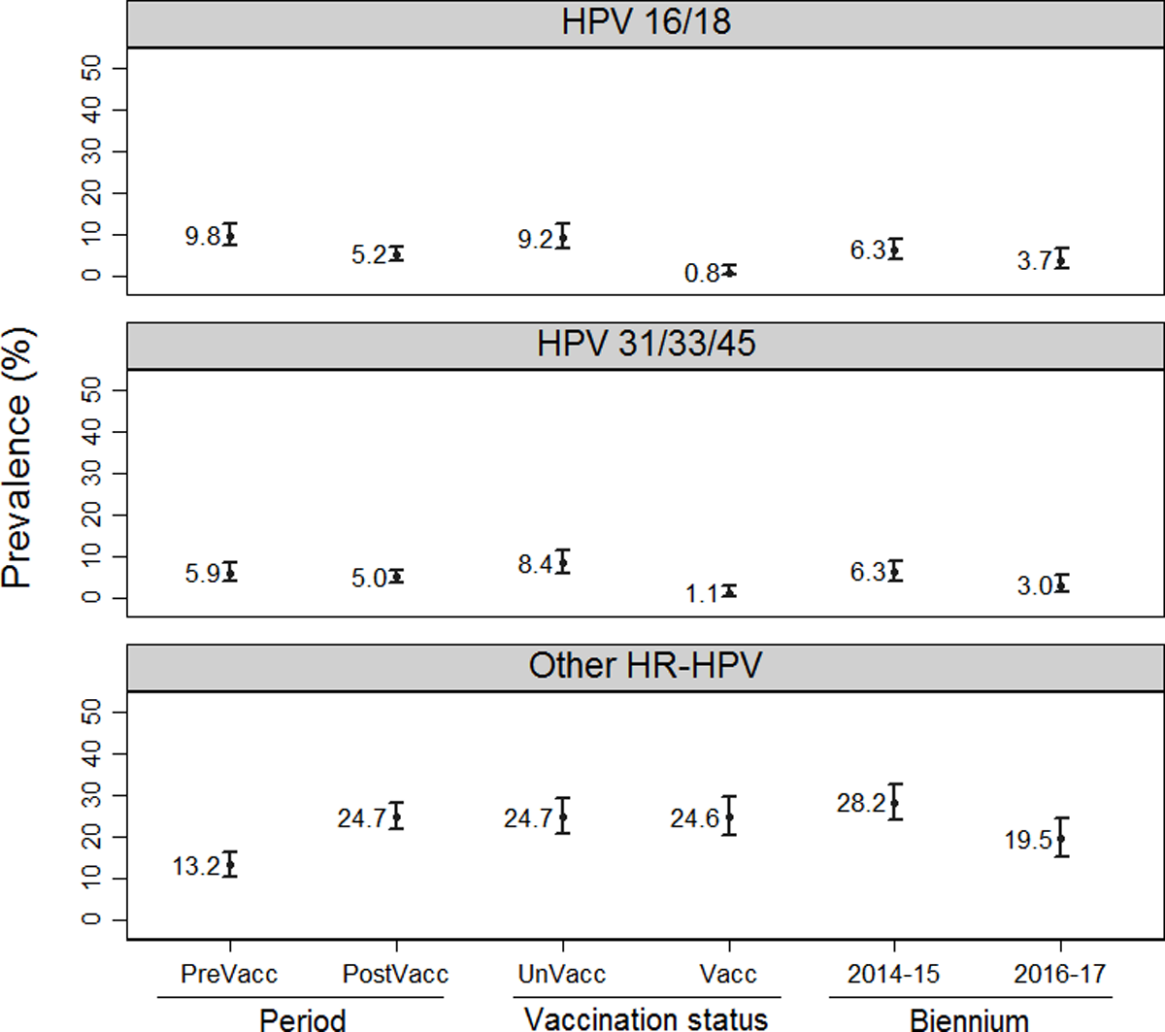
Global HR-HPV group prevalence by period of study. **HR-HPV group prevalence in post-vaccination period by vaccination status and post-vaccination biennium**.

HPV 16/18 was detected by the Cobas® 4800 HPV test (Roche Diagnostics) in 39 (5.2%) samples and other HR-HPV different than 16/18 in 199 (26.7%). Among these 199 samples, 128 (64.3%) were genotyped by the Linear Array® HPV genotyping test (Roche Diagnostics) and the genotype was automatically assigned in the rest of samples as described in the statistical analysis section. The calculated prevalence of three groups of HR-HPV in the studied populations is reported in Fig 1.

### Vaccine effectiveness against HPV 16/18

**Fig 2:**
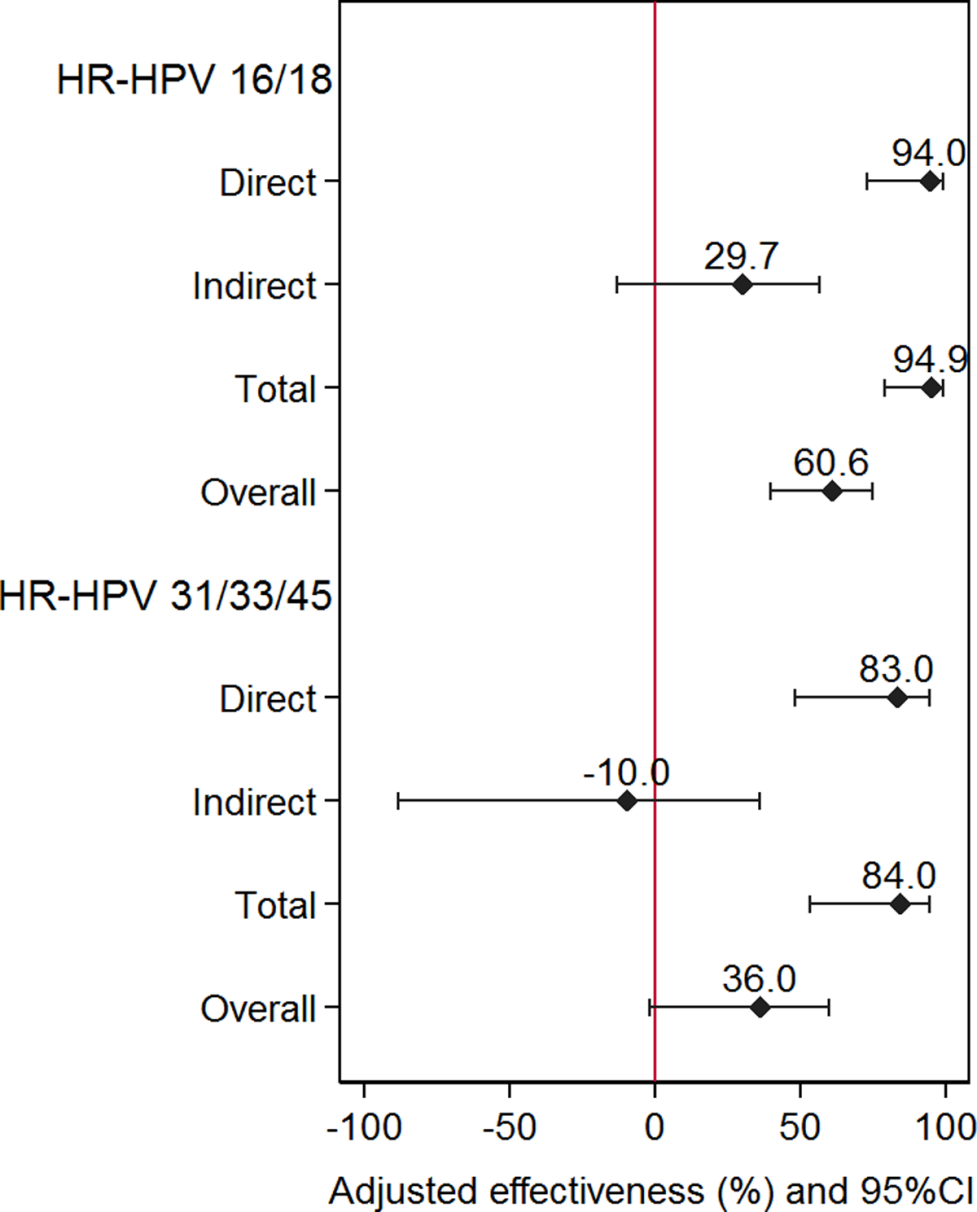
Adjusted effectiveness of HR-HPV 16/18 and 31/33/45, in vaccinated *vs*. unvaccinated women in the post-vaccination period (direct effectiveness), unvaccinated women in the post-vaccination period *vs*. women in the pre-vaccination period (indirect effectiveness), vaccinated women *vs*. women in the pre-vaccination period (total effectiveness), and vaccinated and unvaccinated women in the post-vaccination period *vs*. women in the pre-vaccination period (overall effectiveness).

In the post-vaccination period, 39 women had a positive result for HPV 16/18. The prevalence of HPV 16/18 was 9.2% (95% CI: 6.5−12.5) in unvaccinated women *vs*. 0.8% (95% CI: 0.2−2.5) in vaccinated women (Fig 1), giving rise to an adjusted direct effectiveness of 94% (95% CI: 72−99) (Fig 2 and Table 3). The adjusted total effectiveness was 95% (95% CI: 79−99); and the adjusted overall effectiveness was 61% (95% CI: 39−74). More detailed results are collected in S1–S4 Tables.

**Table 3:** HPV 16/18: Prevalence ratio employed for calculating direct, indirect, total and overall effectiveness.

	PR	95% CI		*p* value
**Vaccinated *vs*. Unvaccinated**				
**Raw**	0.09	0.03	0.30	*< 0.001
**Adjusted**	0.06	0.01	0.28	*< 0.001
**Unvaccinated *vs*. Pre-vaccination period**				
**Raw**	0.94	0.63	1.41	0.772
**Adjusted**	0.70	0.44	1.11	0.130
**Vaccinated *vs*. Pre-vaccination period**				
**Raw**	0.09	0.03	0.28	*< 0.001
**Adjusted**	0.05	0.01	0.21	*< 0.001
**Post *vs*. Pre-vaccination period**				
**Raw**	0.54	0.36	0.80	0.002
**Adjusted**	0.39	0.26	0.61	*< 0.001

PR: Prevalence ratio. CI: Confidence interval.

* *p* < 0.05, statistically significant.

### Vaccine effectiveness against HPV 31/33/45

The prevalence of HPV 31/33/45 was 8.4% (95% CI: 5.9−11.6) for unvaccinated women *vs*. 1.1% (95% CI: 0.3−2.9) for vaccinated women (Fig 1). The adjusted direct effectiveness calculated in this case was 83% (95% CI: 46−94) (Fig 2 and Table 4). The adjusted total effectiveness was 84% (95% CI: 54−94) and adjusted overall effectiveness was 36% (95% CI: -2−60). More detailed results are given in S5–S8 Tables.

**Table 4:** HPV 31/33/45: Prevalence ratio employed for calculating direct, indirect, total and overall effectiveness.

	PR	95% CI		*p* value
**Vaccinated *vs*. Unvaccinated**				
**Raw**	0.13	0.05	0.38	*<0.001
**Adjusted**	0.17	0.06	0.54	*0.002
**Unvaccinated *vs*. Pre-vaccination period**				
**Raw**	1.42	0.89	2.28	0.146
**Adjusted**	1.10	0.67	1.80	0.702
**Vaccinated *vs*. Pre-vaccination period**				
**Raw**	0.19	0.07	0.54	*0.002
**Adjusted**	0.16	0.06	0.46	*0.001
**Post *vs*. Pre-vaccination period**				
**Raw**	0.84	0.53	1.33	0.455
**Adjusted**	0.64	0.40	1.02	0.061

PR: Prevalence ratio. CI: Confidence interval.

* *p* < 0.05, statistically significant.

### Vaccine effectiveness against other HR-HPV genotypes

The prevalence of other HR-HPV is shown in Fig 1. The prevalence of other HR-HPV for vaccinated women and unvaccinated women, was 24.6% (95% CI: 20.2−29.5) and 24.7% (95% CI: 20.6−29.3), respectively, yielding a PR of 1 (Table 5). No significant differences were found between their prevalence ratios for the periods 2014–15 (1.09, 95% CI: 0.78−1.53) and 2016–17 (1.15, 95% CI: 0.66−1.99). More detailed results are shown in S9–S12 Tables.

**Table 5:** Raw and adjusted prevalence ratios of HR-HPV genotypes different from 16/18/31/33/45, in vaccinated *vs*. unvaccinated women, unvaccinated women *vs*. pre-vaccination period, vaccinated women *vs*. pre-vaccination period, and post-vaccination period (vaccinated and unvaccinated women) *vs*. pre-vaccination period.

	PR	95% CI		*p* value
**Vaccinated *vs*. Unvaccinated**				
**Raw**	1.00	0.77	1.28	0.975
**Adjusted**	1.02	0.77	1.35	0.908
**Unvaccinated *vs*. Pre-vaccination period**				
**Raw**	1.88	1.42	2.48	*<0.001
**Adjusted**	1.52	1.14	2.03	*0.004
**Vaccinated *vs*. Pre-vaccination period**				
**Raw**	1.87	1.40	2.49	*<0.001
**Adjusted**	1.73	1.28	2.35	*<0.001
**Post *vs*. Pre-vaccination period**				
**Raw**	1.87	1.45	2.41	*<0.001
**Adjusted**	1.66	1.28	2.14	*<0.001

PR: Prevalence ratio. CI: Confidence interval.

* *p* < 0.05, statistically significant.

## Discussion

Regarding sexual habits changes in the Galician population, it was observed that women included in the post-vaccination period started sexual activity earlier and had a higher number of sexual partners along life than women included in the pre-vaccination period. In addition, analyzing only women included in the post-vaccination period, the youngest women (vaccinated group) were younger at first intercourse than the older women (unvaccinated group). Despite of this, an important decrease in HPV 16/18 prevalence and a high direct immunization effectiveness (94%) was observed in vaccinated women in comparison with unvaccinated women in the same period of time (post-vaccination period).

On the other hand, the estimated unadjusted PR for HPV 16/18 between the post-vaccination and the pre-vaccination period was 0.5 (50% decrease), reaching values previously reported by Markowitz *et al*. [14] or Kavanagh *et al*. [15].

Although unvaccinated women at the post-vaccination period started sexual risk behaviors earlier than women at the pre-vaccination period, the indirect vaccination effectiveness was 29.7% (*p* = 0.145). This suggests a herd immunological effect of this vaccine already observed in other studies by Cameron *et al*. [16] and Tabrizi *et al*. [17]. As expected, the total effectiveness was estimated to be 95%, which we believe, it has not been previously reported. The overall vaccine effectiveness was 61%, as obtained from an adjusted prevalence ratio of 0.39, which is in line with that published by Mesher *et al*. [11].

We also looked for cross-protection in this vaccinated population. The estimated direct and total vaccination effectiveness were 83% and 84%, respectively, for the HPV 31/33/45 group of genotypes. These results agree with the trend already observed by Kavanagh *et al*. [15] in women receiving three vaccine doses and by Cameron *et al*. [16] in those receiving two doses. The cross-protection findings could have clinical consequences in the prevention of precancerous and cancerous lesions related to HPV 31/33/45 [3]. Although these genotypes are not targeted by the vaccine, certain cross-protection was already published [18,19] and explained by their genetic relationship with HPV 16/18. Likewise, the results of this study are consistent with those observed by Drolet *et al*. [20] as well as with the results of Mesher *et al*. [11] obtained for the 16–18 year-old group with an estimated HPV vaccine coverage of 65%; in these studies, there was no direct information about the vaccination status of women included in the target group for vaccination.

On the other hand, the overall vaccine effectiveness was 36%, which seems to indicate that there is a population effect, although the CIs show that is not statistically significant probably due to small sample size. It is remarkable that in other studies [14,17], analyzing the introduction of quadrivalent vaccine, there were not statistically significant differences in the prevalence of genotypes 31/33/45 between pre-vaccination and post-vaccination periods. Unlike the large direct and total effectiveness, indirect effectiveness was not observed for HPV 31/33/45. Reasons explaining this fact may be the sample size and/or the time elapsed since the vaccine introduction.

We have also analyzed the possibility of replacement between HR-HPV genotypes in the post-vaccination period. The same prevalence for other HR-HPV was observed for vaccinated and unvaccinated women, independently of the prevalence of HPV 16/18 and HPV 31/33/45, discarding any replacement between genotypes. In the case that replacement had occurred, one would expect a higher prevalence for vaccinated than for unvaccinated women, but the adjusted prevalence ratio was 1.02. The observed increase in the prevalence of other HR-VPH in the post-vaccination period versus the pre-vaccination period could be explained by the greater presence of risk factors in the post-vaccination period.

Kavanagh *et al*. [15] did not find evidence of clinically significant high-risk type-replacement, although they observed a trend over time for prevalence of any HR-HPV excluding 16/18/31/33/45. This trend depended on birth cohort (more likely to infect the younger cohort) and on the number of administered doses (less likely in case of complete vaccination with three doses). This last finding of Kavanagh was not found in a study published by Cameron *et al*. [16].

Different methods showed good concordance to estimate vaccination coverage, demonstrating good knowledge about the HPV vaccine among the young population. In this study 74% of the women of the cohort from 1994 onwards were vaccinated with three doses. This proportion is very close to the 72% of global vaccination coverage in Galicia (data provided by the Communicable Diseases Department of the Public Health General Direction from Consellería de Sanidade, Galicia, Spain, for birth cohorts from 1994 to 1999). This supports that results of this study are representative of the Galician population. Although 90% of vaccinated women included in this study received three doses, those receiving two doses were also considered as vaccinated. This criterion allowed us to increase the sample size relying on data on two doses vaccination effectiveness observed in other studies [14,21].

This study has several strengths. As stated by Cameron *et al*. [16], there are few studies in which the vaccination status can be directly linked to HPV detection in cervical samples. In addition, microbiological data are accompanied by sexual habits information and the vaccination status was verified by the electronic clinical history. More than 98% of the population have access to free health care. This supports that this study is a good representation of the Galician female population. In our community, given the characteristics of the Public Health System, there is nothing to presume that only a specific subgroup of women had been invited to participate.

Four limitations of this study could be listed: (i) the small sample size; (ii) the impossibility of verifying the external validity of the sample; (iii) the imputation of data on 71 samples; and (iv) the fact that two different methods were used for HPV detection in pre-vaccination and post-vaccination periods, although these two tests have previously shown a very high agreement for the detection of HR-HPV genotypes [22].

In conclusion, this study highlights the achievements of the vaccination program followed in our community. A high direct effectiveness previously described for HPV 16/18 was also supported by this study. A possibly relevant result is the high direct cross effectiveness (83%) shown for some other genotypes (HPV 31/33/45) not targeted by the bivalent vaccine. An indirect effectiveness of 30% was observed for genotypes 16/18; although statistical significance was not achieved with our sample size, the information could be of interest, as it supports an expected result not yet demonstrated. Our results seem to indicate that there was no common HPV replacement by other types. Continuous surveillance importance lies not only in the evaluation of vaccine effectiveness but also in the future assessment of genotype replacement or cross protection persistence along time.

## Supporting information

S1 Appendix Sample size calculation. (DOCX)

S2 Appendix Pre-vaccination questionnaire. (DOCX)

S3 Appendix Post-vaccination questionnaire. (DOCX)

S1 Table Prevalence ratio (PR) for HR-HPV 16/18 and 95% CI in vaccinated vs. unvaccinated women in the post-vaccination period. (DOC)

S2 Table Prevalence ratio (PR) for HR-HPV 16/18 and 95% CI in unvaccinated women in the post-vaccination period vs. women in the pre-vaccination period. (DOC)

S3 Table Prevalence ratio (PR) for HR-HPV 16/18 and 95% CI in vaccinated women in the post-vaccination period vs. women in the pre-vaccination period. (DOC)

S4 Table Prevalence ratio (PR) for HR-HPV 16/18 and 95% CI in vaccinated and unvaccinated women in the post-vaccination period *vs*. women in the pre-vaccination period. (DOCX)

S5 Table Prevalence ratio (PR) for HR-HPV 31/33/45 and 95% CI in vaccinated vs. unvaccinated women in the post-vaccination period. (DOC)

S6 Table Prevalence ratio (PR) for HR-HPV 31/33/45 and 95% CI in unvaccinated women in the post-vaccination period vs. women in the pre-vaccination period. (DOC)

S7 Table Prevalence ratio (PR) for HR-HPV 31/33/45 and 95% CI in vaccinated women in the post-vaccination period vs. women in the pre-vaccination period. (DOC)

S8 Table Prevalence ratio (PR) for HR-HPV 31/33/45 and 95% CI in vaccinated and unvaccinated women in the post-vaccination period *vs*. women in the pre-vaccination period. (DOCX)

S9 Table Prevalence ratio (PR) for HR-HPV excluding 16/18/31/33/45 and 95% CI in vaccinated vs. unvaccinated women in the post-vaccination period. (DOC)

S10 Table Prevalence ratio (PR) for HR-HPV excluding 16/18/31/33/45 and 95% CI in unvaccinated women in the post-vaccination period vs. women in the pre-vaccination period. (DOC)

S11 Table Prevalence ratio (PR) for HR-HPV excluding 16/18/31/33/45 and 95% CI in vaccinated women in the post-vaccination period vs. women in the pre-vaccination period. (DOC)

S12 Table Prevalence ratio (PR) for HR-HPV excluding 16/18/31/33/45 and 95% CI in vaccinated and unvaccinated women in the post-vaccination period *vs*. women in the pre-vaccination period. (DOCX)
