# Supplementary material for: Direct, indirect and total effectiveness of bivalent HPV vaccine in women in Galicia, Spain
Source: PLoS One. 2018 Aug 3;13(8):e0201653. doi: 10.1371/journal.pone.0201653 (PMC6075752; doi:10.1371/journal.pone.0201653)
Supplement: S1 Appendix — (DOCX) [file pone.0201653.s001.docx]

Sample size was calculated based on an expected prevalence of 2.9% for HPV 16/18 in women aged 20 - 25 years, and an error between 1.5% and 2%. The expected prevalence was estimated from the pre-vaccination prevalence in Galicia (10%), the expected vaccination coverage (80%), facilitated by the Galician Vaccination Program (Dirección Xeral de Saúde Pública, Consellería de Sanidade, Galicia, Spain) and the effectiveness of the bivalent vaccine (90%) described in the literature [1, 2]. It resulted in a size of 1260 women for each of the two established groups.

The agreed action strategy was to include 60 women per year (30 per group) in each of the seven Health Areas for a period of six years. This publication presents the first results obtained from July of 2014 to July of 2017.

References:

1. World Health Organization. Cervical cancer, human papillomavirus (HPV), and HPV vaccines - Key points for policy-makers and health professionals [Internet]. WHO Press, World Health Organization; 2007. Available: [http://apps.who.int/iris/bitstream/10665/69873/1/WHO_RHR_08.14_eng.pdf](http://apps.who.int/iris/bitstream/10665/69873/1/WHO_RHR_08.14_eng.pdf#_blank)

2. Bruni L, Serrano B, Bosch X, Castellsagué X. Human papillomavirus vaccine. Efficacy and safety. Enferm Infecc Microbiol Clin. 2015;33: 342–354. doi:10.1016/j.eimc.2015.03.018
