# Supplementary material for: Direct, indirect and total effectiveness of bivalent HPV vaccine in women in Galicia, Spain
Source: PLoS One. 2018 Aug 3;13(8):e0201653. doi: 10.1371/journal.pone.0201653 (PMC6075752; doi:10.1371/journal.pone.0201653)
Supplement: S2 Appendix — (DOCX) [file pone.0201653.s002.docx]

**Pre-vaccination questionnaire (Spanish/English)**

En este cuestionario hay ciertas preguntas que usted puede considerar de carácter íntimo, pero se pueden contestar con total sinceridad porque de ningún modo se sabrá quién contestó a este cuestionario.

In this questionnaire there are certain questions that you may consider of an intimate nature, but you can answer them with total sincerity because nobody will know who answered this questionnaire.

(1) ¿Qué día nació?________________ (día/mes/año) When were you born? _____ (day / month / year)

(2) ¿Dónde nació? Where were you born?

En Galicia In Galicia

Fuera de Galicia Out of Galicia

Si nació fuera de Galicia ¿en qué país nació? If you were born out of Galicia, Which country were you born in? _____________

¿En qué año llegó a Galicia? Which year did you arrive in Galicia?____________

(3) En la actualidad ¿qué tipo anticonceptivos usa? Currently, what type of contraception do you use?

No uso/ none  Pastillas/ pills  DIU/ IUD  Preservativo?/condom  otro tipo/other

(4) ¿Cuantos años tenía cuando tuvo la primera relación sexual completa (es decir, con penetración)?_____ años. How old were you when you had the first complete sexual intercourse (that is with penetration)? _____ years old.

(5) En esa ocasión, ¿utilizó preservativo? On that occasion, did you use a condom?  Si/Yes  No/Not

(5) Y, desde entonces, ¿con cuantas parejas distintas tuvo relaciones sexuales completas (es decir, con penetración)?_____ And, since then, how many different partners have you had complete sexual intercourse with (that is, with penetration)? _____

(6) Si tiene relaciones sexuales ocasionales....If you have casual sex…

No tengo relaciones sexuales ocasionales / I do not have casual sex

Siempre son con preservativo/ I always use a condom

Casi siempre son con preservativo/ I use a condom ocassionally.

Casi nunca son con preservativo / I hardly ever use condom

Nunca son con preservativo /I never use a condom

(7) Concentrándonos ahora en el último año/ Concentrating now in the last year:

¿Ha tenido relaciones sexuales completas (es decir con penetración)? Have you had complete sex (that is with penetration)?

No/ No

Si, ¿con cuantas parejas distintas?__Yes, with how many different partners ?:___
