# Supplementary material for: Direct, indirect and total effectiveness of bivalent HPV vaccine in women in Galicia, Spain
Source: PLoS One. 2018 Aug 3;13(8):e0201653. doi: 10.1371/journal.pone.0201653 (PMC6075752; doi:10.1371/journal.pone.0201653)
Supplement: S3 Appendix — (DOCX) [file pone.0201653.s003.docx]

**Post-vaccination questionnaire (Spanish/English)**

En este cuestionario hay ciertas preguntas que usted puede considerar de carácter íntimo, pero se pueden contestar con total sinceridad porque de ningún modo se sabrá quién contestó a este cuestionario.

In this questionnaire there are certain questions that you may consider of an intimate nature, but you can answer them with total sincerity because nobody will know who answered this questionnaire.

(1) ¿Qué día nació? ____________(día/mes/año) When were you born? _____ (day / month / year)

(2) ¿Dónde nació? Where were you born?

En Galicia In Galicia.

Fuera de Galicia Out of Galicia.

Si nació fuera de Galicia: ¿en qué país nació? If you were born out of Galicia, Which country were you born in? _____________

¿En qué año llegó a Galicia? Which year did you arrive in Galicia?:____________

(3) ¿Cuantos años tenía cuando tuvo la primera relación sexual completa (es decir, con penetración)?_____ años. How old were you when you had the first complete sexual intercourse (that is with penetration)? _____ years old.

(4) Y, desde entonces, ¿con cuantas parejas distintas, ocasionales o duraderas, tuvo relaciones sexuales completas (es decir, con penetración)?: And, since then, how many different casual or long-term partners have you had complete sexual intercourse with (that is, with penetration)?_____parejas/ partners.

(5) Concentrándonos ahora en el último año: Concentrating now in the last year:

¿Ha tenido relaciones sexuales completas (es decir con penetración)? Have you had complete sex intercourse (that is with penetration)?

No/ No

Si, ¿con cuantas parejas distintas? ____Yes, with how many different partners?___

(6) Recibió la vacuna frente al Virus del Papiloma humano que se pone con 14 o más años de edad?

Did you receive the vaccine against the Human Papillomavirus at 14 or more years old?

Si/ yes

No/ No

No lo sé/ I don´t know.

(7) Solo si ha recibido la vacuna, ¿cuantas dosis recibió? In case you have been vaccinated, how many doses did you receive?

1

2

3

No lo sé/ I don´t know.

(8) Sólo si recibió la vacuna, ¿qué edad tenía cuando recibió la última dosis? In case you have received the vaccine, how old were you when you received the last dose? _____años/ years old.
