# Supplementary material for: Direct, indirect and total effectiveness of bivalent HPV vaccine in women in Galicia, Spain
Source: PLoS One. 2018 Aug 3;13(8):e0201653. doi: 10.1371/journal.pone.0201653 (PMC6075752; doi:10.1371/journal.pone.0201653)
Supplement: S1 Table — (DOC) [file pone.0201653.s004.doc]

**S1 Table. Prevalence ratio (PR) for HR-HPV 16/18 and 95% CI in vaccinated vs. unvaccinated women in the post-vaccination period.**

|  | **PR** | **95% CI** | | ***p* value** |
| --- | --- | --- | --- | --- |
| **Raw** |  |  |  |  |
| **Vaccinated (*vs.* Unvaccinated)** | 0.09 | 0.03 | 0.30 | *< 0.001 |
| **Adjusted** |  |  |  |  |
| **Vaccinated** | 0.06 | 0.01 | 0.28 | *< 0.001 |
| **21-23 years old (*vs*. 18 – 20)** | 0.85 | 0.32 | 2.29 | 0.755 |
| **24-26 years old (*vs*. 18 – 20)** | 0.65 | 0.23 | 1.89 | 0.434 |
| **Age at first intercourse > 16** | 0.88 | 0.42 | 1.83 | 0.725 |
| **Three or more partners along life** | 1.58 | 0.68 | 3.66 | 0.290 |
| **Two or more partners in the last year** | 1.24 | 0.62 | 2.47 | 0.543 |

PR: Prevalence ratio. CI: Confidence interval. * p < 0.05, statistically significant.
