# Supplementary material for: Direct, indirect and total effectiveness of bivalent HPV vaccine in women in Galicia, Spain
Source: PLoS One. 2018 Aug 3;13(8):e0201653. doi: 10.1371/journal.pone.0201653 (PMC6075752; doi:10.1371/journal.pone.0201653)
Supplement: S2 Table — (DOC) [file pone.0201653.s005.doc]

**S2 Table. Prevalence ratio (PR) for HR-HPV 16/18 and 95% CI in unvaccinated women in the post-vaccination period vs. women in the pre-vaccination period.**

|  | **PR** | **95% CI** | | ***p* value** |
| --- | --- | --- | --- | --- |
| **Raw** |  |  |  |  |
| **Unvaccinated (*vs*. Pre-vaccination period)** | 0.94 | 0.63 | 1.41 | 0.772 |
| **Adjusted** |  |  |  |  |
| **Unvaccinated** | 0.70 | 0.45 | 1.11 | 0.130 |
| **21 – 23 years old (*vs*. 18 – 20)** | 1.09 | 0.63 | 1.89 | 0.761 |
| **24 – 26 years old (*vs*. 18 – 20)** | 1.02 | 0.58 | 1.77 | 0.957 |
| **Age at first intercourse > 16** | 0.81 | 0.52 | 1.26 | 0.347 |
| **Three or more partners along life** | 1.85 | 1.10 | 3.12 | *0.020 |
| **Two or more partners in the last year** | 1.49 | 0.97 | 2.29 | 0.071 |

PR: Prevalence ratio. CI: Confidence interval. * *p* < 0.05, statistically significant.
