# Supplementary material for: Direct, indirect and total effectiveness of bivalent HPV vaccine in women in Galicia, Spain
Source: PLoS One. 2018 Aug 3;13(8):e0201653. doi: 10.1371/journal.pone.0201653 (PMC6075752; doi:10.1371/journal.pone.0201653)
Supplement: S3 Table — (DOC) [file pone.0201653.s006.doc]

**S3 Table. Prevalence ratio (PR) for HR-HPV 16/18 and 95% CI in vaccinated women in the post-vaccination period vs. women in the pre-vaccination period.**

|  | **PR** | **95% CI** | | ***p* value** |
| --- | --- | --- | --- | --- |
| **Raw** |  |  |  |  |
| **Vaccinated (*vs*. Pre-vaccination period)** | 0.09 | 0.03 | 0.28 | *< 0.001 |
| **Adjusted** |  |  |  |  |
| **Vaccinated** | 0.05 | 0.01 | 0.21 | *< 0.001 |
| **21 – 23 years old (*vs*. 18 – 20)** | 1.11 | 0.57 | 2.16 | 0.753 |
| **24 – 26 years old (*vs*. 18 – 20)** | 1.13 | 0.60 | 2.11 | 0.710 |
| **Age at first intercourse > 16** | 0.83 | 0.48 | 1.42 | 0.489 |
| **Three or more partners along life** | 2.46 | 1.29 | 4.69 | *0.006 |
| **Two or more partners in the last year** | 1.36 | 0.79 | 2.32 | 0.269 |

PR: Prevalence ratio. CI: Confidence interval. * *p* < 0.05, statistically significant.
