# Supplementary material for: Direct, indirect and total effectiveness of bivalent HPV vaccine in women in Galicia, Spain
Source: PLoS One. 2018 Aug 3;13(8):e0201653. doi: 10.1371/journal.pone.0201653 (PMC6075752; doi:10.1371/journal.pone.0201653)
Supplement: S4 Table — (DOCX) [file pone.0201653.s007.docx]

**S4 Table. Prevalence ratio (PR) for HR-HPV 16/18 and 95% CI in vaccinated and unvaccinated women in the post-vaccination period *vs.* women in the pre-vaccination period.**

|  | **PR** | **95% CI** | | ***p* value** |
| --- | --- | --- | --- | --- |
| **Raw** |  |  |  |  |
| **Post-vaccination period (vs. Pre-vaccination period)** | 0.54 | 0.36 | 0.80 | 0.002 |
| **Adjusted** |  |  |  |  |
| **Post-vaccination period** | 0.39 | 0.26 | 0.61 | *<0.001 |
| **21 – 23 years old (*vs*. 18 – 20)** | 1.46 | 0.86 | 2.45 | 0.157 |
| **24 – 26 years old (*vs*. 18 – 20)** | 1.50 | 0.87 | 2.57 | 0.144 |
| **Age at first intercourse > 16** | 0.81 | 0.52 | 1.26 | 0.349 |
| **Three or more partners along life** | 2.01 | 1.20 | 3.39 | 0.009 |
| **Two or more partners in the last year** | 1.37 | 0.89 | 2.12 | 0.152 |

PR: Prevalence ratio. CI: Confidence interval. * p < 0.05, statistically significant.
