# Supplementary material for: Direct, indirect and total effectiveness of bivalent HPV vaccine in women in Galicia, Spain
Source: PLoS One. 2018 Aug 3;13(8):e0201653. doi: 10.1371/journal.pone.0201653 (PMC6075752; doi:10.1371/journal.pone.0201653)
Supplement: S5 Table — (DOC) [file pone.0201653.s008.doc]

**S5 Table. Prevalence ratio (PR) for HR-HPV 31/33/45** and 95% CI in vaccinated vs. unvaccinated women in the post-vaccination period.

|  | **PR** | **95% CI** | | ***p* value** |
| --- | --- | --- | --- | --- |
| **Raw** |  |  |  |  |
| **Vaccinated (*vs.* Unvaccinated)** | 0.13 | 0.05 | 0.38 | *<0.001 |
| **Adjusted** |  |  |  |  |
| **Vaccinated** | 0.17 | 0.06 | 0.54 | *0.002 |
| **21 – 23 years old (*vs*. 18 – 20)** | 1.91 | 0.53 | 6.89 | 0.323 |
| **24 – 26 years old (*vs*. 18 – 20)** | 1.60 | 0.40 | 6.33 | 0.506 |
| **Age at first intercourse > 16** | 0.95 | 0.49 | 1.84 | 0.879 |
| **Three or more partners along life** | 4.32 | 1.22 | 15.26 | *0.023 |
| **Two or more partners in the last year** | 3.72 | 1.87 | 7.41 | *<0.001 |

PR: Prevalence ratio. CI: Confidence interval. * *p* < 0.05, statistically significant.
