# Supplementary material for: Direct, indirect and total effectiveness of bivalent HPV vaccine in women in Galicia, Spain
Source: PLoS One. 2018 Aug 3;13(8):e0201653. doi: 10.1371/journal.pone.0201653 (PMC6075752; doi:10.1371/journal.pone.0201653)
Supplement: S6 Table — (DOC) [file pone.0201653.s009.doc]

**S6 Table. Prevalence ratio (PR) for HR-HPV 31/33/45 and 95% CI in unvaccinated women in the post-vaccination period vs. women in the pre-vaccination period.**

|  | **PR** | **95% CI** | | ***p* value** |
| --- | --- | --- | --- | --- |
| **Raw** |  |  |  |  |
| **Unvaccinated (*vs*. Pre-vaccination period)** | 1.42 | 0.89 | 2.28 | 0.146 |
| **Adjusted** |  |  |  |  |
| **Unvaccinated** | 1.10 | 0.67 | 1.80 | 0.702 |
| **21 – 23 years old (*vs*. 18 – 20)** | 1.14 | 0.60 | 2.17 | 0.696 |
| **24 – 26 years old (*vs*. 18 – 20)** | 0.94 | 0.48 | 1.83 | 0.850 |
| **Age at first intercourse > 16** | 1.04 | 0.64 | 1.68 | 0.883 |
| **Three or more partners along life** | 2.92 | 1.44 | 5.92 | *0.003 |
| **Two or more partners in the last year** | 2.82 | 1.73 | 4.59 | *<0.001 |

PR: Prevalence ratio. CI: Confidence interval. * *p* <0.05, statistically significant.
