# Supplementary material for: Direct, indirect and total effectiveness of bivalent HPV vaccine in women in Galicia, Spain
Source: PLoS One. 2018 Aug 3;13(8):e0201653. doi: 10.1371/journal.pone.0201653 (PMC6075752; doi:10.1371/journal.pone.0201653)
Supplement: S7 Table — (DOC) [file pone.0201653.s010.doc]

**S7 Table. Prevalence ratio (PR) for HR-HPV 31/33/45 and 95% CI in vaccinated women in the post-vaccination period vs. women in the pre-vaccination period.**

|  | **PR** | **95% CI** | | ***p* value** |
| --- | --- | --- | --- | --- |
| **Raw** |  |  |  |  |
| **Vaccinated (*vs*. Pre-vaccination period)** | 0.19 | 0.07 | 0.54 | *0.002 |
| **Adjusted** |  |  |  |  |
| **Vaccinated** | 0.16 | 0.06 | 0.46 | 0.001 |
| **21 – 23 years old (*vs*. 18 – 20)** | 1.19 | 0.56 | 2.54 | 0.648 |
| **24 – 26 years old (*vs*. 18 – 20)** | 1.17 | 0.52 | 2.63 | 0.707 |
| **Age at first intercourse > 16** | 0.78 | 0.41 | 1.49 | 0.455 |
| **Three or more partners along life** | 1.84 | 0.78 | 4.32 | 0.164 |
| **Two or more partners in the last year** | 2.74 | 1.36 | 5.52 | *0.005 |

PR: Prevalence ratio. CI: Confidence interval. * *p* < 0.05, statistically significant.
