# Supplementary material for: Direct, indirect and total effectiveness of bivalent HPV vaccine in women in Galicia, Spain
Source: PLoS One. 2018 Aug 3;13(8):e0201653. doi: 10.1371/journal.pone.0201653 (PMC6075752; doi:10.1371/journal.pone.0201653)
Supplement: S8 Table — (DOCX) [file pone.0201653.s011.docx]

**S8 Table. Prevalence ratio (PR) for HR-HPV 31/33/45 and 95% CI in vaccinated and unvaccinated women in the post-vaccination period *vs.* women in the pre-vaccination period.**

|  | **PR** | **95% CI** | | ***p* value** |
| --- | --- | --- | --- | --- |
| **Raw** |  |  |  |  |
| **Post-vaccination period (vs. Pre-vaccination period)** | 0.84 | 0.53 | 1.33 | 0.455 |
| **Adjusted** |  |  |  |  |
| **Post-vaccination period** | 0.64 | 0.40 | 1.02 | 0.061 |
| **21 – 23 years old (*vs*. 18 – 20)** | 1.71 | 0.94 | 3.09 | 0.077 |
| **24 – 26 years old (*vs*. 18 – 20)** | 1.68 | 0.89 | 3.15 | 0.107 |
| **Age at first intercourse > 16** | 0.90 | 0.56 | 1.46 | 0.674 |
| **Three or more partners along life** | 2.69 | 1.34 | 5.39 | 0.005 |
| **Two or more partners in the last year** | 2.91 | 1.77 | 4.78 | *<0.001 |

PR: Prevalence ratio. CI: Confidence interval. * p < 0.05, statistically significant.
