# Supplementary material for: Direct, indirect and total effectiveness of bivalent HPV vaccine in women in Galicia, Spain
Source: PLoS One. 2018 Aug 3;13(8):e0201653. doi: 10.1371/journal.pone.0201653 (PMC6075752; doi:10.1371/journal.pone.0201653)
Supplement: S9 Table — (DOC) [file pone.0201653.s012.doc]

**S9 Table. Prevalence ratio (PR) for HR-HPV excluding 16/18/31/33/45** and 95% CI in vaccinated vs. unvaccinated women in the post-vaccination period.

|  | **PR** | **95% CI** | | ***p* value** |
| --- | --- | --- | --- | --- |
| **Raw** |  |  |  |  |
| **Vaccinated (*vs.* Unvaccinated)** | 1.00 | 0.77 | 1.28 | 0.975 |
| **Adjusted** |  |  |  |  |
| **Vaccinated** | 1.02 | 0.77 | 1.35 | 0.908 |
| **21 – 23 years old (*vs*. 18 – 20)** | 0.91 | 0.66 | 1.25 | 0.573 |
| **24 – 26 years old (*vs*. 18 – 20)** | 0.93 | 0.63 | 1.35 | 0.689 |
| **Age at first intercourse > 16** | 1.09 | 0.84 | 1.42 | 0.514 |
| **Three or more partners along life** | 1.71 | 1.24 | 2.36 | *0.001 |
| **Two or more partners in the last year** | 2.00 | 1.55 | 2.59 | *<0.001 |

PR: Prevalence ratio. CI: Confidence interval. * *p* < 0.05, statistically significant.
