# Supplementary material for: Direct, indirect and total effectiveness of bivalent HPV vaccine in women in Galicia, Spain
Source: PLoS One. 2018 Aug 3;13(8):e0201653. doi: 10.1371/journal.pone.0201653 (PMC6075752; doi:10.1371/journal.pone.0201653)
Supplement: S10 Table — (DOC) [file pone.0201653.s013.doc]

**S10 Table. Prevalence ratio (PR) for HR-HPV excluding 16/18/31/33/45 and 95% CI in unvaccinated women in the post-vaccination period vs. women in the pre-vaccination period.**

|  | **PR** | **95% CI** | | ***p* value** |
| --- | --- | --- | --- | --- |
| **Raw** |  |  |  |  |
| **Unvaccinated (*vs*. Pre-vaccination period)** | 1.88 | 1.42 | 2.48 | *<0.001 |
| **Adjusted** |  |  |  |  |
| **Unvaccinated** | 1.52 | 1.14 | 2.03 | *0.004 |
| **21 – 23 years old (*vs*. 18 – 20)** | 1.08 | 0.73 | 1.61 | 0.697 |
| **24 – 26 years old (*vs*. 18 – 20)** | 1.19 | 0.80 | 1.76 | 0.396 |
| **Age at first intercourse > 16** | 1.03 | 0.78 | 1.35 | 0.853 |
| **Three or more partners along life** | 2.46 | 1.66 | 3.65 | *<0.001 |
| **Two or more partners in the last year** | 2.13 | 1.61 | 2.81 | *<0.001 |

PR: Prevalence ratio. CI: Confidence interval. * *p* < 0.05, statistically significant.
