# Supplementary material for: Direct, indirect and total effectiveness of bivalent HPV vaccine in women in Galicia, Spain
Source: PLoS One. 2018 Aug 3;13(8):e0201653. doi: 10.1371/journal.pone.0201653 (PMC6075752; doi:10.1371/journal.pone.0201653)
Supplement: S11 Table — (DOC) [file pone.0201653.s014.doc]

**S11 Table. Prevalence ratio (PR) for HR-HPV excluding 16/18/31/33/45 and 95% CI in vaccinated women in the post-vaccination period vs. women in the pre-vaccination period.**

|  | **PR** | **95% CI** | | ***p* value** |
| --- | --- | --- | --- | --- |
| **Raw** |  |  |  |  |
| **Vaccinated (*vs*. Pre-vaccination period)** | 1.87 | 1.40 | 2.49 | *<0.001 |
| **Adjusted** |  |  |  |  |
| **Vaccinated** | 1.73 | 1.28 | 2.35 | *<0.001 |
| **21 – 23 years old (*vs*. 18 – 20)** | 0.94 | 0.69 | 1.30 | 0.721 |
| **24 – 26 years old (*vs*. 18 – 20)** | 1.08 | 0.73 | 1.59 | 0.707 |
| **Age at first intercourse > 16** | 1.03 | 0.76 | 1.40 | 0.846 |
| **Three or more partners along life** | 1.92 | 1.36 | 2.72 | *<0.001 |
| **Two or more partners in the last year** | 1.85 | 1.38 | 2.48 | *<0.001 |

PR: Prevalence ratio. CI: Confidence interval. * p < 0.05, statistically significant.
