# Supplementary material for: Direct, indirect and total effectiveness of bivalent HPV vaccine in women in Galicia, Spain
Source: PLoS One. 2018 Aug 3;13(8):e0201653. doi: 10.1371/journal.pone.0201653 (PMC6075752; doi:10.1371/journal.pone.0201653)
Supplement: S12 Table — (DOCX) [file pone.0201653.s015.docx]

**S12 Table. Prevalence ratio (PR) for HR-HPV excluding 16/18/31/33/45 and 95% CI in vaccinated and unvaccinated women in the post-vaccination period *vs.* women in the pre-vaccination period.**

|  | **PR** | **95% CI** | | ***p* value** |
| --- | --- | --- | --- | --- |
| **Raw** |  |  |  |  |
| **Post-vaccination period (vs. Pre-vaccination period)** | 1.87 | 1.45 | 2.41 | *<0.001 |
| **Adjusted** |  |  |  |  |
| **Post-vaccination period** | 1.66 | 1.28 | 2.14 | *<0.001 |
| **21 – 23 years old (*vs*. 18 – 20)** | 0.94 | 0.73 | 1.22 | 0.652 |
| **24 – 26 years old (*vs*. 18 – 20)** | 1.01 | 0.76 | 1.34 | 0.936 |
| **Age at first intercourse > 16** | 1.05 | 0.84 | 1.32 | 0.662 |
| **Three or more partners along life** | 1.97 | 1.48 | 2.63 | *<0.001 |
| **Two or more partners in the last year** | 2.00 | 1.60 | 2.50 | *<0.001 |

PR: Prevalence ratio. CI: Confidence interval. * p < 0.05, statistically significant.
